# Impact of the model of long‐term follow‐up care on adherence to guideline‐recommended surveillance among survivors of adolescent and young adult cancers

**DOI:** 10.1002/cam4.4058

**Published:** 2021-06-15

**Authors:** Dalia Kagramanov, Rinku Sutradhar, Cindy Lau, Zhan Yao, Jason D. Pole, Nancy N. Baxter, Sumit Gupta, Paul C. Nathan

**Affiliations:** ^1^ University of Toronto Institute of Medical Science Toronto Canada; ^2^ University of Toronto Institue of Health Policy Management and Evaluation Toronto Canada; ^3^ Centre for Health Services Research The University of Queensland Brisbane Australia; ^4^ Division of Hematology/Oncology The Hospital for Sick Children Toronto Canada; ^5^ Keenan Research Center of the Li Ka Shing Knowledge Institute of St. Michael's Hospital Toronto Canada; ^6^ University of Toronto Mount Sinai Hospital Toronto Canada; ^7^ ICES Toronto Canada

**Keywords:** adolescent and young adult, cancer survivor, follow‐up care, surveillance, survivorship

## Abstract

**Purpose:**

Adolescent and young adult cancer survivors require lifelong healthcare to address the late effects of therapy. We examined the impact of different provider models of long‐term follow‐up (LTFU) care on adherence to recommended surveillance.

**Methods:**

We conducted a retrospective cohort study using administrative health databases in Ontario, Canada. Five‐year survivors were identified from IMPACT, a database of patients aged 15–20.9 years at diagnosis of six cancers between 1992 and 2010. We defined three models of LTFU care hierarchically: specialized survivor clinics (SCCs), general cancer clinics (GCCs), and family physician (FP). We assessed adherence to the Children's Oncology Group surveillance guidelines for cardiomyopathy and breast cancer. Multistate models assessed adherence transitions and impacts of LTFU attendance.

**Results:**

A total of 1574 survivors were followed for a mean of 9.2 years (range 4.3–13.9 years) from index (5‐year survival). The highest level of LTFU attended in the first 2‐years post‐index was a GCC (47%); only 16.7% attended a SCC. By the end of study, 72% no longer attended any of the models of care and only 2% still attended an SCC. Among 188 survivors requiring breast cancer surveillance, 6.9% were adherent to their first required surveillance testing. Attendance at a SCC in the previous year and higher cumulative FP or GCC visits increased the rate of subsequently becoming adherent. Among 857 survivors requiring cardiomyopathy surveillance, 11% were adherent at study entry. Each subsequent SCC visit led to an 11.3% (95% CI: 1.05–1.18) increase in the rate of becoming adherent.

**Conclusion:**

LTFU attendance and surveillance adherence are sub‐optimal. SCC follow‐up is associated with greater adherence, but few survivors receive such care, and this proportion diminished over time. Interventions are needed to improve LTFU attendance and promote surveillance adherence.

## Graphical abstract

Long‐term follow‐up care attendance and surveillance adherence are sub‐optimal among adolescent and young adult cancer survivors. Specialized survivor clinic follow‐up is associated with greater adherence, but few survivors receive such care, and this proportion diminished over time. Interventions are needed to improve the long‐term follow‐up care attendance and promote surveillance adherence.

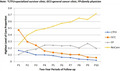

## 1 INTRODUCTION

Although cancer is the leading cause of disease‐related death among adolescents and young adults (AYA) aged 15–29,^1^ improvements in therapy and supportive care have resulted in over 80% of AYA diagnosed with cancer becoming long‐term survivors.^2^ Survivors are at an elevated risk for developing chronic physical and psychological morbidities (late effects) that can impact both the quality and duration of their life.^3^, ^4^ As a result, lifelong healthcare focused on each survivor's specific risks has been advocated.^5^ Organizations such as the North American Children's Oncology Group (COG) have published surveillance guidelines for the late effects of cancer therapy^6^ such as subsequent malignancies and cardiac dysfunction.

Several models have been proposed for the delivery of long‐term follow‐up care (LTFU) to survivors of AYA cancers. These vary by provider (e.g., oncologist, primary‐care physician such as a family doctor or internist, nurse practitioner) and location (specialized survivor clinic vs. general cancer clinic vs. family physician office).^7^ The optimal LTFU model has not yet been established.^8^ While family physicians are more accessible to most survivors, they frequently lack specialized knowledge and comfort relevant to this population.^9^

In Ontario, Canada, the Ministry of Health and Long‐term Care funds a network of specialized multidisciplinary survivor clinics intended to provide lifelong risk‐based care to cancer survivors diagnosed prior to age 18 years. However, access is conditional on having been treated at a pediatric institution. Since AYA diagnosed with cancer can receive therapy at a pediatric center, adult cancer center, or community hospital, these specialized survivor clinics are not accessible to all AYA survivors. Survivors in Ontario who do not attend a specialized clinic may receive LTFU care from an oncologist in a general cancer clinic in an adult cancer center or community hospital, from their FP, or have no LTFU care at all. Information on the impact of LTFU care models on adherence to surveillance in AYA survivors is minimal; some prior work in survivors of childhood cancers in Ontario has suggested that attendance at SCCs had a strong association with adherence to cardiomyopathy screening compared to no attendance.^10^

To investigate LTFU attendance and its relationship with adherence to surveillance further, we linked provincial cancer registries to administrative health databases in Ontario to determine the models of LTFU care accessed by this population of AYA cancer survivors and to understand whether location of survivor care is related to adherence to recommended surveillance.

## 2 METHODS

This retrospective cohort study was approved by Research Ethics Boards at the Hospital for Sick Children and Sunnybrook Health Sciences Centre. The cohort was identified from the Initiative to Maximize Progress in Adolescent and Young Adult Cancer Therapy (IMPACT), an Ontario provincial AYA cohort^11^ of patients aged 15–21 years at diagnosis of one of six prevalent AYA cancers (leukemia, Hodgkin Lymphoma, non‐Hodgkin lymphoma, testicular cancer, bone sarcoma, or soft‐tissue sarcoma) between 1992 and 2010. Eligible survivors had survived at least 5 years from initial cancer diagnosis. Survivors identified in IMPACT were linked to provincial health administrative databases, described in Table S1 (Online Only) using a unique, encoded identifier. These databases are housed at ICES, a non‐for‐profit research institute that holds an array of Ontario's health‐related data. Data sharing is not applicable to this paper as no datasets were generated or analyzed for this current study.

### 2.1 *Follow*‐*up period*

Survivors were followed from an index date, defined as 5 years from their primary cancer diagnosis. If survivors experienced a subsequent malignant neoplasm [SMN], relapse, or progression within 5 years of diagnosis, the index date was re‐defined as 5 years after the most recent event. The occurrence of relapse or progression of disease or development of an SMN was captured by IMPACT. Survivors were observed from index until the earliest of the end of study (31 December 2016), death, or any cancer event occurring more than 5 years from the initial cancer diagnosis.

### 2.2 Exposure

We classified LTFU care into three categories: (i) *Specialized* *survivor* *clinics*––one of five SCCs for adult survivors of childhood cancer; (ii) *General* *cancer* *clinic––*care at an adult cancer center or community hospital by an oncologist/hematologist; and (iii) Family Physician––visits to a primary care physician were considered as follow‐up if they consisted of a full history and physical examination, as defined in previous studies,^12^, ^13^ using the Ontario Health Insurance Plan (OHIP) billing codes listed in Appendix S1 (online only). OHIP, a database of all physician billing claims in Ontario since 1991, was used to identify and track the utilization of physician services at each of the levels of LTFU care.

### 2.3 Outcome measures

Adherence to surveillance among AYA survivors was defined according to the COG guidelines (Version 4.0). Survivors at risk for cardiomyopathy or breast cancer based on chemotherapy and radiation exposures^6^ were previously captured and calculated in IMPACT. The criteria for defining risk, as well as the recommended frequency of surveillance testing, are presented in Table 1. OHIP billing codes (listed in Appendix S1, online only) identified the occurrences of breast imaging (mammogram, breast MRI, and ultrasound) and echocardiograms. Periods of follow‐up were created based on each survivor's recommended surveillance according to the COG. As of 2003, when the COG follow‐up guidelines were created, breast cancer surveillance was recommended annually beginning at the later of age of 25 or 8 years after therapy or diagnosis for females that received chest radiation. Cardiac surveillance was recommended every 1, 2, or 5 years depending on the risk. Baseline adherence to breast cancer surveillance and cardiomyopathy was defined as 1 year preceding the date each survivor required surveillance according to the COG guidelines. The relationship between each category of LTFU and adherence was analyzed using the following variables: LTFU attendance in the previous year, and cumulative attendance at each level of care over time.

**TABLE 1:** Surveillance guidelines for survivors at risk for breast cancer and cardiomyopathy

Breast cancer surveillance guidelines			
Breast radiation exposure		Surveillance guidelines	
≥20 Gy radiation therapy to the chest		Annual mammography starting 8 years after radiation or age 25 years, whichever is last	
Cardiomyopathy surveillance guidelines			
Age at treatment	Radiation with potential impact to the heart	Anthracycline dose	Recommended frequency
<1 year old	Yes	Any	Every year
	No	<200 mg/m^2^	Every 2 years
1–4 years old	Yes	Any	Every year
	No	≥100 to <300 mg/m^2^	Every 2 years
		≥300 mg/m^2^	Every year
>5 years old	Yes	<300 mg/m^2^	Every 2 years
		≥300 mg/m^2^	Every year
	No	≥200 mg/m^2^ to <300 mg/m^2^	Every 2 years
		≥300 mg/m^2^	Every year
Any age with decrease in serial function			Every year

### 2.4 Covariates

Baseline patient characteristics included sex and age at diagnosis. Treatment‐related information included primary diagnosis, location of first cancer treatment (pediatric cancer center vs. regional cancer center vs. adult community hospital), receipt of chemotherapy, radiation or hematopoietic stem‐cell transplant (each classified as “yes”/“no”), and occurrences of SMN, relapse, or progression of disease. Socioeconomic status (SES) was divided into quintiles of neighborhood deprivation using ONMARG (a database that quantifies SES using neighborhood data on residential instability, material deprivation, dependency, and ethnic concentration^14^) and rurality was categorized into urban or rural. Distances to the closest specialized survivor clinic and/or general cancer clinic were calculated using a straight line distance from each survivor's residence. SES, rurality, and distance variables were captured for all survivors at index and updated annually to be incorporated into the regression models as time‐varying covariates.

### 2.5 Statistical analysis

All analyses were performed with SAS for Unix (Version 9.3). Continuous variables were reported using mean and standard deviation, while dichotomous variables were reported as counts and percentages. Annual periods of follow‐up were created for each individual starting at the index date until the earliest of a censoring event or the end of the study in order to assess a crude representation of LTFU care visits to each model.

A multistate modeling framework was developed to examine adherence to surveillance over time.^15^, ^16^, ^17^ Since survivor adherence status can vary widely over the course of follow‐up, a multistate model was used to better reflect the natural back and forth transitions of survivors between adherence and non‐adherence. We designated survivors as being at risk for breast cancer and/or cardiomyopathy using their cancer treatment data and the COG guidelines for screening and analyzed each at‐risk group separately. Consistent with prior work examining screening adherence,^18^, ^19^, ^20^ the multistate model consisted of three states: *State* *1*: *adherent*; *State 2*: *non*‐*adherent*; and *State 3*: *dead*, *relapse*, *or* *SMN (after* *index)*. States 1 and 2 were non‐absorbing states as transitions could be made back and forth between them. State 3 was an absorbing state as no further transitions were possible after this point. We modeled the instantaneous rate/intensity of transition from one state to another, and determined factors associated with each transition rate.^21^ Under this 3‐state model, univariable regression for each transition rate was first conducted for all predictor variables. Backward selection with *p* value cut‐off <0.1 was used to include variables in the multivariable regression for each transition rate, where LTFU care was retained as the main exposure during the backward selection process.

## 3 RESULTS

The cohort consisted of 1574 AYA cancer survivors, 508 (32.3%) treated for their cancer at a pediatric center and 1066 (67.7%) treated at an adult cancer center or community hospital. Median follow‐up time from index was 9.2 years (range 4.3–13.9 years). Approximately two thirds of the cohort was male (62.4%), with an equal distribution of patients diagnosed in each age group between 15 and 20.9 years of age. Baseline characteristics for all survivors are summarized in Table 2.

**TABLE 2:** Baseline characteristics of AYA survivors

Predictor	Category	*N*	% of Cohort
Total		1574	100
Sex	Male	982	62.4
	Female	592	37.6
Age at diagnosis	15–15.9	215	13.6
	16–16.9	252	16
	17–17.9	287	18.2
	18–18.9	289	18.3
	19–19.9	250	15.8
	20–20.9	281	17.8
Primary diagnosis	Hodgkin lymphoma	641	40.7
	Non‐Hodgkin lymphoma	191	12.1
	Leukemia‐AML +Other	83	5.3
	Leukemia ‐ALL	151	9.6
	Soft‐tissue sarcoma	124	7.9
	Bone sarcoma	119	7.6
	Testicular cancer	265	16.8
Treatment location of first cancer	Pediatric cancer center	508	32.3
	Adult cancer center	886	56.3
	Community hospital	180	11.4
Chemotherapy	Yes	1197	76.1
	No	377	23.9
Radiation	Head + Brain	118	7.5
	Neck	431	27.4
	Thorax	488	31
	Abdomen + Pelvis	214	13.6
	Other	198	12.6
	No	920	58.4
Hematopoietic stem‐cell transplant prior to index	Autologous	69	4.3
	Allogeneic	68	4.3
	No	1439	91.4
SMN prior to index^b^	Yes	10	0.6
	No	1564	99.4
Relapse/progression prior to index	Yes	175	11.1
	No	1399	88.9
Socioeconomic status (SES)^a^	1 low deprivation	272	17.3
	2	297	18.9
	3	320	20.3
	4	338	21.5
	5 high deprivation	335	21.3
	Unknown	12	0.8
Rurality^a^	Yes	167	10.6
	No	1403	89.4

^a^ Covariate was updated over time but presented in the table at baseline only.

^b^ SMN = Second malignant neoplasms.

### 3.1 Frequency of LTFU care attendance

**FIGURE 1:**
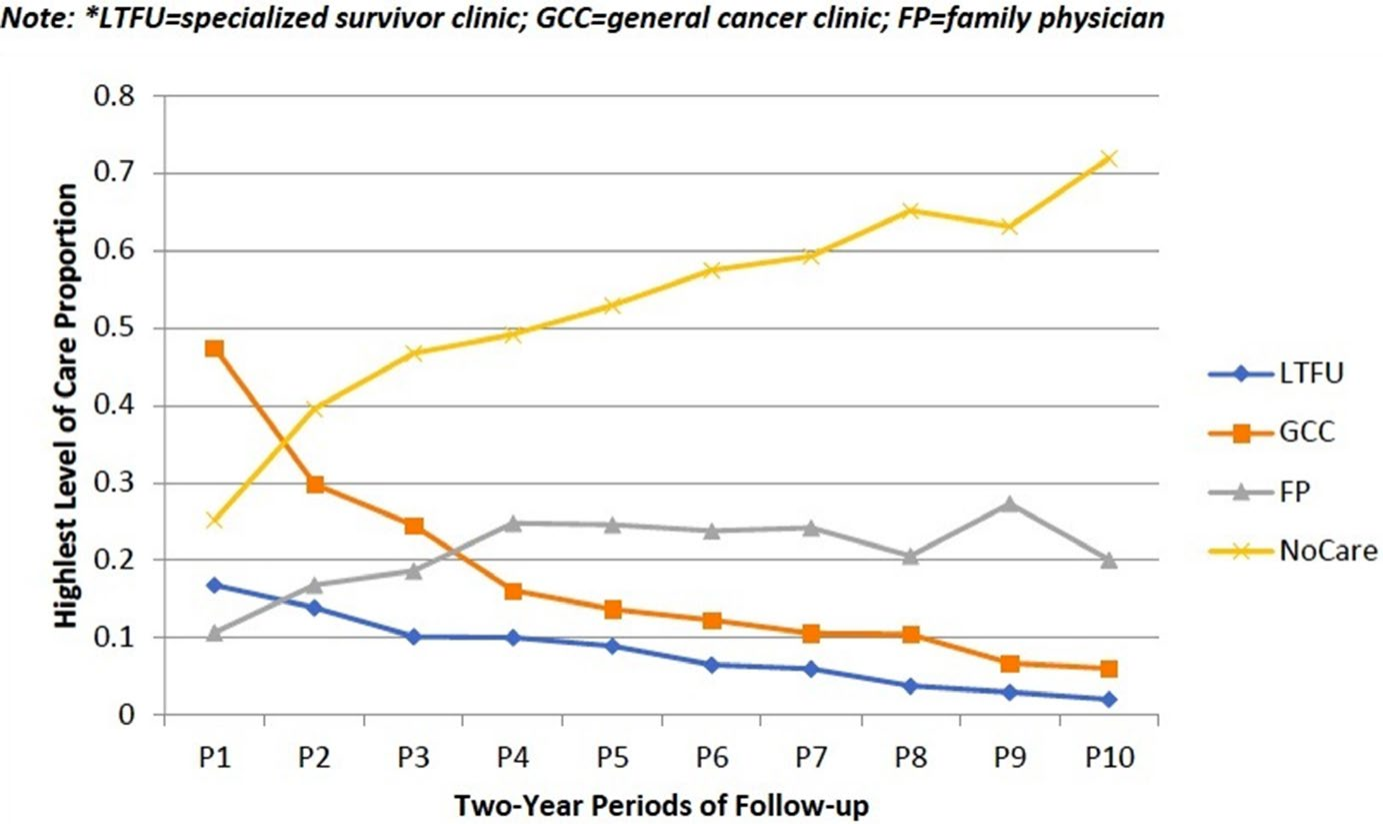
Proportion of patients accessing long‐term follow‐up care care per period of follow‐up (highest level of care shown)/LTFU = specialized survivor care, GCC = general cancer clinic, FP = family physician

To assess LTFU attendance hierarchically, the proportion of survivors accessing each level of care within each 2‐year period since index was calculated. Among all survivors irrespective of their initial cancer treatment location, the highest level of care within the first 2 years after index was a general cancer clinic among 47.3%, followed by a specialized survivor clinic (16.7%) and a FP (9.3%). The remaining 26.7% had no identified survivor care during this period (Figure 1). At the end of follow‐up, a median of 8.7 years from index (range 4.3–13.9), the highest level of care with the preceding 2 years was a general cancer clinic in 6%, a specialized survivor clinic in 2%, and a FP in 20%. Seventy‐two percent were receiving no follow‐up care.

### 3.2 Breast cancer surveillance

There were 188 women who required breast cancer screening according to the COG guidelines. At baseline (up to 1 year prior to the date each survivor required), 13 (6.9%) survivors were adherent and 175 (93.1%) were non‐adherent. Of those not adherent at baseline, 84/175 (48.0%) remained non‐adherent throughout. None of the 13 survivor's adherent at baseline remained adherent throughout follow‐up. Multivariable predictors of changing adherence states are presented in Table 3 (Table S2 displays rates of attendance across models of care, while Tables S4 and S5 display univariable results). Each 1‐year increase in age from the time they required surveillance lowered the rate of a survivor becoming non‐adherent by 61% (RR 0.39, 95% CI 0.29–0.54, *p*<0.001), while each additional visit to a specialized survivor clinic decreased the rate of a survivor becoming non‐adherent by 9% (RR 0.91, 95% CI 0.87–0.96, *p*<0.0001). Moreover, attendance at a specialized survivor clinic in the previous year (RR 1.97, 95% CI 1.29–3.01, *p*<0.002), cumulative FP visits (RR 1.10, 95% CI 1.04–1.17, *p*<0.001), and cumulative general cancer clinic visits (RR 1.07, 95% CI 1.04–1.09, *p*<0.0001) increased the rate of a survivor becoming adherent from a non‐adherent state.

**TABLE 3:** Multivariable multistate modeling regression analysis for survivors at risk of breast cancer (statistically significant results)

Parameter	Level	Rate of becoming adherent				Rate of becoming non‐adherent			
		RR	95% LCL	95% UCL	*p*<0.05	RR	95% LCL	95% UCL	*p*<0.05
Age	Age at follow‐up start					0.398	0.291	0.543	**<0.0001**
Specialized clinic cumulative						0.909	0.869	0.951	**<0.0001**
Specialized clinic previous 1 year	Yes versus No	1.971	1.289	3.014	**0.002**				
PCP cumulative		1.104	1.044	1.167	**0.001**	1.032	0.996	1.070	0.086
PCP previous 1 year	Yes versus No	1.301	0.972	1.740	0.077	0.795	0.622	1.016	0.067
General cancer clinic cumulative		1.066	1.043	1.089	**<0.0001**				
General cancer clinic previous 1 year	Yes versus No					0.775	0.598	1.004	0.054
Treatment before index	Chemo and chest rad versus chest rad only	0.726	0.498	1.057	0.095	1.275	1.016	1.601	**0.036**

Bolded values are statistically significant (p<0.05).

## 4 Cardiomyopathy surveillance

An analysis of cardiomyopathy surveillance was conducted for survivors requiring annual or biennial imaging. Eight hundred and fifty‐seven survivors required such screening, of whom 94 (11.0%) were adherent at baseline. Over the course of the study, 15/94 (16.0%) adherent at baseline remained adherent throughout, while 598/763 (78.3%) who were non‐adherent at baseline remained non‐adherent. Table 4 presents the results of the multivariable analysis of transition state predictors for survivors at risk for cardiomyopathy, requiring surveillance annually or biennially (Tables S6 and S7 display univariable results). Survivors who received anthracycline chemotherapy (with or without radiation to the chest) had a higher rate (RR 1.75, 95% CI 1.21–2.54, *p*<0.003) of remaining adherent compared to survivors who received radiation to the chest but no anthracyclines. Moreover, survivors who had a specialized survivor clinic visit in the previous year (RR 1.60, 95% CI 1.02–2.52, *p*<0.042) showed a greater rate of becoming adherent from a previously non‐adherent state. Cumulative counts of prior specialized survivor clinic attendance had the largest impact on the rate of becoming adherent (RR 1.11, 95% CI 1.05–1.18), with cumulative visits to general cancer clinics (RR 1.03, 95% CI 1.00–1.05) and FPs (RR 1.07, 95% CI 1.01–1.13) having significant but smaller impacts.

**TABLE 4:** Multivariable multistate modeling regression analysis for survivors at risk of cardiomyopathy (1&2 year surveillance frequency) (statistically significant results)

Parameter	Level	Rate of becoming adherentent				Rate of becoming non‐adher			
		RR	95% LCL	95% UCL	*p*<0.05	RR	95%LCL	95%UCL	*p*<0.05
Age	Age at follow‐up start	0.906	0.838	0.980	**0.014**				
Cardiac surveillance frequency	2 year versus 1 year					0.429	0.363	0.507	**<0.001**
Specialized clinic cumulative		1.113	1.048	1.181	**0.001**				
Specialized clinic previous 1 year	Y versus N	1.602	1.016	2.524	**0.042**	0.472	0.363	0.614	**<0.0001**
PCP cumulative		1.066	1.010	1.126	**0.021**				
General cancer clinic cumulative		1.026	1.004	1.047	**0.017**	0.977	0.964	0.990	**0.001**
SES	2 versus 1	1.026	0.688	1.530	0.899				
	3 versus 1	0.550	0.352	0.860	**0.009**				
	4 versus 1	0.703	0.470	1.053	0.088				
	5 versus 1	0.818	0.550	1.217	0.321				
Sex	F versus M	1.375	1.020	1.853	**0.037**				
Treatment before index	Chemo and chest rad versus chemo only	1.753	1.208	2.544	**0.003**				

Bolded values are statistically significant (p<0.05).

## 5 DISCUSSION

In this population‐based study of the 1574 survivors of AYA cancer, almost three‐quarters had no regular source of LTFU care by the end of the study, an average of 9 years from entering survivorship. Moreover, a concerningly low proportion of survivors was adherent to guideline‐recommended surveillance for late effects of cancer therapy. By the end of the study, only half of at‐risk survivors were adherent to breast cancer surveillance. Sixteen percent of those that required annual echocardiography and 48% of those who required biennial echocardiography were adherent. These low adherence rates indicate that most survivors are not being monitored appropriately for late effects, decreasing their chances of earlier detection and improved outcome.

The most common location of follow‐up care in the first 2 years after entering survivorship was at a general cancer clinic, suggesting that most 5‐year survivors who remain in active care are still initially engaged with the clinic where they received their cancer therapy. Survivor preference is a critical factor in determining where follow‐up care is received. A recent Swiss study revealed that AYA survivors rated follow‐up care from the medical oncologist who provided initial therapy higher than all other models of care, including attendance at a multidisciplinary survivor clinic, visits to a general practitioner, or follow‐up by telephone/questionnaires.^22^ Similarly, a US study demonstrated that the majority of AYA cancer survivors preferred to receive LTFU care from their primary oncologist with whom they already had a close relationship.^23^ However, GCCs usually care for a mix of on‐treatment and off‐treatment patients, may focus less on monitoring for late effects of therapy, and frequently do not have a multidisciplinary team with specific expertise in survivorship. These observations are supported by our data which showed that even though survivors were more likely to attend GCCs, specialized clinic attendance lead to greater adherence to breast and cardiac surveillance guidelines.

Among the survivors in our study, FP visits accounted for the highest proportion of LTFU care attendance over time. However, without being provided with appropriate information about a survivor's prior treatment, future risks, and recommended surveillance, FPs may not tailor their history, physical exam, and counseling to a survivor's prior cancer. A report from the North America Childhood Cancer Survivor Study (CCSS) revealed that only 17.8% of survivors who saw a FP reported receiving care that included advice on how to reduce their risk for late effects or the discussion/ordering of screening tests.^23^ A survey of 1124 FPs across the United States and Canada revealed that only 33%, 27%, and 23% of respondents felt comfortable caring for survivors of the Hodgkin Lymphoma, ALL, or osteosarcoma, respectively. Furthermore, only 16% and 10% could correctly identify the appropriate guideline‐recommended surveillance for survivors at risk for breast cancer and cardiomyopathy, respectively.^9^ Many FPs express discomfort caring for AYA cancer survivors^9^ and as a result, appropriate referrals may not be made for surveillance according to the guideline recommendations.

Despite the location or provider of follow‐up care, rates of surveillance were low for both breast cancer and cardiac screening. The CCSS recently reported on 8522 survivors and demonstrated poor adherence to surveillance among survivors at high risk for breast, skin, colorectal cancer, and cardiac disease.^24^ In our study, the majority of survivors did not have a regular source of LTFU care; however, among survivors who attended one of the models of LTFU care, cardiomyopathy surveillance was less likely among survivors receiving care in a general cancer clinic or from a FP compared to care from a specialized clinic. Although adherence to surveillance recommendations was generally low irrespective of LTFU attendance, it is notable that cumulative general cancer clinic and FP visits were associated with a 3% and 7% greater probability per visit of becoming adherent, respectively, while cumulative specialized survivor clinic attendance resulted in an 11% greater probability per visit of becoming adherent. These findings are consistent with a prior study of adult survivors of childhood cancer in Ontario which showed that survivors who attended a specialized clinic had a 10.6 times greater rate of adherence to annual guideline‐recommended screening for cardiomyopathy when compared to no attendance.^10^

Unfortunately, although all types of follow‐up care have a positive impact on surveillance, low attendance rates translate into majority survivors not being surveilled appropriately. In our study, only one third of the cohort was eligible to attend a specialized survivor clinic since these are restricted to AYA treated at a pediatric cancer center. Despite having access to a specialized survivor clinic, few survivors attended this model of survivor care. Prior research has shown that other modifiers of attendance include distance to a specialized clinic (as these are all located in large urban centers), age at initial cancer diagnosis, and location of initial cancer therapy.^25^ Barriers to appropriate LTFU care, regardless of where such care is provided, include lack of knowledge, cost, wishing to move on with life, competing life responsibilities, lower education levels and lower perceived levels of social support.^26^, ^27^ A study conducted in 2016 revealed that AYA cancer survivors have a more positive perception of their health compared to healthy controls,^28^ a potential contributing factor to not seeking regular health care. Future work should focus on patient education of risks and surveillance, as well the empowerment to seek regular care as a survivor.

There is no perfect LTFU model. Our results demonstrate that sustained attendance at specialized clinics or GCCs decreases over time. A deeper understanding of the elements of SCCs that positively drive surveillance, and application of these elements to other care models that survivors are more likely to attend, such as a FP, may be beneficial to improving LTFU care. Research has shown that despite gaps in knowledge, FPs are generally willing to care for the childhood cancer survivor population if given specific tools such as patient‐specific letters, survivor‐care plans, or working in collaboration with a cancer center.^9^

Our findings should be interpreted in the context of several limitations. First, we focused on a cohort located in a single province in Canada, which may limit the generalizability of our results, since not all jurisdictions have the same hierarchy of models available. However, SCCs exist across Canada, as well as in many countries (although not all countries provide universal access to health care, and health insurance status is likely an important determinant of health care access^29^). Second, our young AYA cohort was aged 15–21 years of age at diagnosis, while AYA has been variably defined in the literature to include adults up to the age of 39. However, the young age of our cohort encompasses many life transitions such as graduation, moving away from home, and transferring to adult care providers, suggesting that this is a particularly vulnerable group of AYA. Lastly, it was not possible to know exactly what occurred during each captured LTFU visit and whether this was representative of risk‐based care. Particularly for visits to a general cancer clinic or FP, we could not be sure that the content of the visits focused on the survivor's prior cancer and their risk for late effects.

The results from this study, in conjunction with prior literature, add clarity to our understanding of LTFU care in AYA cancer survivors as well as the impact of LTFU care on adherence to guideline‐recommended surveillance. The understanding that adherence to guideline recommended surveillance is sub‐optimal and a majority of survivors have no regular source of follow‐up care by the end of study, provides information on opportunities to improve the lifelong care of survivors of AYA cancer.

## CONFLICT OF INTEREST

None.

## ETHICS APPROVAL

This retrospective cohort study was approved by Research Ethics Boards at the Hospital for Sick Children and Sunnybrook Health Sciences Centre.

## Supporting information

Table S1‐S7‐Appendix‐S1

## Data Availability

Data sharing is not applicable to this article as no new data were created or analyzed in this study.
