# Supplementary material for: Impact of the model of long‐term follow‐up care on adherence to guideline‐recommended surveillance among survivors of adolescent and young adult cancers
Source: Cancer Med. 2021 Jun 15;10(15):5078–87. doi: 10.1002/cam4.4058 (PMC8335842; doi:10.1002/cam4.4058)
Supplement: Supplementary file 1 — Table S1‐S7‐Appendix‐S1 [file CAM4-10-5078-s001.docx]

**SUPPLEMENTAL FILES**

***Supplemental Table 1: Healthcare Administrative Databases***

| **Database** | **Data Elements** | **Use in Study** |
| --- | --- | --- |
| IMPACT | Demographic information  Diagnosis  Cancer therapies | Create cohort  Collect predictor variables |
| POGONIS | Demographic information  Diagnosis  Cancer therapies | Create cohort  Collect predictor variables |
| OHIP | Diagnostic codes  Fee codes  Location of services  Physician specialty  Adult cancer therapy | Outcome definition  Family physician visits  Emergency room visits 1992-2002  Determine adult relapses |
| NACRS | Diagnostic codes associated with emergency room visits | Collect predictor variables  Emergency room visits 2002-2016 |
| CIHI-DAD | Diagnostic codes associated with hospitalizations | Outcome definition  Hospitalizations 1992-2016 |
| RPDB | Demographic information | Outcome definition |
| IPDB | Physician demographics and specialty | Outcome definition |
| CPDB | Physician service information | Outcome definition |
| CHF | Information on patients with CHF in Ontario | Outcome definition  Collect information on CHF events |
| ONMARG | Quantifies marginalization in Ontario | Collect demographic predictor variables |
| OCR | New incidences of adult cancers | Determine adult cancer diagnoses and/or second cancers |
| ORGD | Death dates and cause of deaths | Study exclusions or patient censoring |
| OBSP | Breast cancer screening dates | Outcome definition |

***Supplemental Table 2: Rates of Attendance Across Models of LTFU Care***

| **Parameter** | **Class** | **Reference** | **RR** | **95% LCL** | **95% UCL** | **P<0.05** | |
| --- | --- | --- | --- | --- | --- | --- | --- |
| Sex | F | Male | 1.35 | 1.214 | 1.501 | **<.0001** |  |
| Treatment Location | Pediatric | Acute Cancer Clinic | 1.21 | 1.029 | 1.422 | **0.0208** |  |
| Cancer Type | Bone Sarcoma | Leukemia | 1.208 | 0.966 | 1.512 | 0.0982 |  |
|  | Hodgkin Lymphoma | Leukemia | 0.864 | 0.709 | 1.054 | 0.1492 |  |
|  | Non-Hodgkin Lymphoma | Leukemia | 0.767 | 0.607 | 0.969 | **0.0262** |  |
|  | Soft-tissue Sarcoma | Leukemia | 0.783 | 0.612 | 1.002 | 0.0517 |  |
|  | Testis | Leukemia | 0.867 | 0.675 | 1.112 | 0.2611 |  |
| Distance to Nearest Specialized Clinic or General Cancer Center | 2 (10-25KM) | 1 | 1.042 | 0.923 | 1.175 | 0.5077 |  |
|  | 3 (25-50KM) | 1 | 0.953 | 0.843 | 1.077 | 0.4387 |  |
|  | 4 (50-100KM) | 1 | 0.949 | 0.824 | 1.092 | 0.4651 |  |
|  | 5 (>100KM) | 1 | 1.013 | 0.815 | 1.258 | 0.9082 |  |
| Chemotherapy | 1 | 0 | 0.921 | 0.785 | 1.08 | 0.3105 |  |
| Radiation | 1 | 0 | 0.855 | 0.647 | 1.13 | 0.2705 |  |
| Chemo & Rad | 1 | 0 | 1.14 | 0.868 | 1.495 | 0.3462 |  |
| BMT | 1 | 0 | 0.811 | 0.647 | 1.017 | 0.0699 |  |
| Age* |  |  | 1.006 | 0.968 | 1.045 | 0.7724 |  |
| Prior # of Visits * |  |  | 1.21 | 1.029 | 1.422 | **0.0208** |  |

****TD=time-dependent variable***

***Supplemental Table 3: Rate of Visits to Specialized Survivor Clinics (Among Survivors Treated Initially at a Pediatric Cancer Center)***

| **Parameter** | **Class** | **RR** | **95% LCL** | **95% UCL** | **P<0.05** |  |
| --- | --- | --- | --- | --- | --- | --- |
| Sex | F | 1.259 | 1.079 | 1.468 | **0.0034** | |
| Cancer Type | Bone Sarcoma | 1.208 | 0.926 | 1.577 | 0.1637 | |
|  | Hodgkin Lymphoma | 1.269 | 1.046 | 1.539 | **0.0157** | |
|  | Non-Hodgkin Lymphoma | 0.921 | 0.676 | 1.255 | 0.6016 | |
|  | Soft-tissue Sarcoma | 0.474 | 0.296 | 0.76 | **0.0019** | |
|  | Testis | 0.113 | 0.026 | 0.49 | **0.0036** | |
| Distance to Nearest Specialized Survivor Clinic | 2 (10-25KM) | 0.681 | 0.558 | 0.831 | **0.0002** | |
|  | 3 (25-50KM) | 0.879 | 0.726 | 1.065 | 0.1869 | |
|  | 4 (50-100KM) | 0.841 | 0.668 | 1.06 | 0.1421 | |
|  | 5 (>100KM) | 0.546 | 0.408 | 0.732 | **<.0001** | |
| Chemo | 1 | 1.063 | 0.706 | 1.602 | 0.7694 | |
| Radiation | 1 | 2.328 | 1.293 | 4.19 | **0.0048** | |
| Chemo + Rad | 1 | 0.622 | 0.335 | 1.158 | 0.1345 | |
| BMT | 1 | 0.41 | 0.238 | 0.706 | **0.0013** | |
| Anthracycline Dose | 1-199 | 0.759 | 0.234 | 2.462 | 0.6457 | |
|  | 200-299 | 1.291 | 0.678 | 2.456 | 0.4367 | |
|  | 300+ | 0.864 | 0.466 | 1.602 | 0.6428 | |
| Age (TD*) |  | 0.954 | 0.869 | 1.048 | 0.3304 | |
| Prior # of Visits (TD*) |  | 1.296 | 1.244 | 1.351 | **<.0001** | |

****TD=time-dependent***

***Supplemental Table 4: Univariable Analysis for Transition State Adherent to Non-Adherent for Breast Cancer Risk***

| **Parameter** | **Class** | **RR** | **95% LCL** | **95% UCL** | **P<0.1** |
| --- | --- | --- | --- | --- | --- |
| Age | Age at Follow-up Start | 0.491 | 0.378 | 0.638 | **<.0001** |
| Distance Nearest Specialized Survivor Clinic |  | 1.000 | 1.000 | 1.000 | 0.975 |
| Distance Nearest General Cancer Clinic |  | 1.000 | 0.996 | 1.005 | 0.872 |
| Specialized Survivor Clinic Cumulative |  | 0.977 | 0.944 | 1.012 | 0.194 |
| Specialized Survivor Clinic Pre-1Yr | Yes vs No | 0.818 | 0.616 | 1.086 | 0.164 |
| Family Physician Cumulative |  | 0.984 | 0.961 | 1.008 | 0.189 |
| Family Physician Pre-1Yr | Yes vs No | 0.860 | 0.705 | 1.050 | 0.139 |
| General Cancer Clinic Cumulative |  | 0.989 | 0.976 | 1.002 | 0.108 |
| General Cancer Clinic Pre-1Yr | Yes vs No | 0.870 | 0.725 | 1.045 | 0.136 |
| Rurality | Urban vs Rural | 0.794 | 0.623 | 1.012 | **0.062** |
| SES | 2 vs 1 | 0.941 | 0.687 | 1.289 | 0.707 |
|  | 3 vs 1 | 0.803 | 0.586 | 1.100 | 0.172 |
|  | 4 vs 1 | 0.807 | 0.580 | 1.121 | 0.201 |
|  | 5 vs 1 | 0.919 | 0.670 | 1.261 | 0.601 |
| Treatment before Index | Chemo and Chest Rad vs. Chest Rad only | 1.079 | 0.930 | 1.251 | 0.317 |

***Supplemental Table 5: Univariable Analysis for Transition State Non-Adherent to Adherent for Breast Cancer Risk***

| **Parameter** | **Class** | **RR** | **95% LCL** | **95% UCL** | **P<0.1** |
| --- | --- | --- | --- | --- | --- |
| Age | Age at Follow-up Start | 0.858 | 0.581 | 1.265 | 0.439 |
| Distance Nearest Specialized Survivor Clinic |  | 1.000 | 1.000 | 1.001 | 0.342 |
| Distance Nearest General Cancer Clinic |  | 0.998 | 0.992 | 1.004 | 0.504 |
| Specialized Survivor Clinic Cumulative |  | 1.100 | 1.030 | 1.175 | **0.004** |
| Specialized Survivor Clinic Pre-1Yr | Yes vs No | 2.289 | 1.514 | 3.460 | **<.0001** |
| Family Physician Cumulative |  | 1.184 | 1.113 | 1.259 | **<.0001** |
| Family Physician Pre-1Yr | Yes vs No | 1.773 | 1.313 | 2.395 | **0.000** |
| General Cancer Clinic Cumulative |  | 1.063 | 1.037 | 1.091 | **<.0001** |
| General Cancer Clinic Pre-1Yr | Yes vs No | 1.762 | 1.237 | 2.509 | **0.002** |
| Rurality | Urban vs Rural | 1.686 | 0.791 | 3.595 | 0.176 |
| SES | 2 vs 1 | 1.022 | 0.575 | 1.816 | 0.941 |
|  | 3 vs 1 | 1.099 | 0.642 | 1.882 | 0.731 |
|  | 4 vs 1 | 1.086 | 0.618 | 1.909 | 0.774 |
|  | 5 vs 1 | 1.201 | 0.739 | 1.951 | 0.459 |
| Treatment before Index | Chemo and Chest Rad vs. Chest Rad only | 0.659 | 0.433 | 1.002 | **0.051** |

***Supplemental Table 6: Univariable Analysis for Transition State Adherent to Non Adherent for Cardiac Cancer Risk (1 & 2 Year Surveillance Combined)***

|  |  |  | | | |  |
| --- | --- | --- | --- | --- | --- | --- |
| **Parameter** | **Class** |  | **RR** | **95% LCL** | **95% UCL** | **P<0.1** |
| Age | Age at Follow-up Start |  | 1.138 | 1.086 | 1.193 | **<.0001** |
| BMT | Yes vs No |  | 1.095 | 0.790 | 1.517 | 0.585 |
| Cardiac Risk Year | 2 years vs 1 year |  | 0.359 | 0.305 | 0.423 | **<.0001** |
| Chemotherapy | Yes vs No |  | 1.367 | 0.992 | 1.883 | **0.056** |
| Chemotherapy + Radiation | Yes vs No |  | 0.850 | 0.661 | 1.093 | 0.206 |
| Distance Nearest Specialized Survivor Clinic |  |  | 1.000 | 1.000 | 1.001 | **0.044** |
| Distance Nearest General Cancer Clinic |  |  | 1.000 | 0.999 | 1.002 | 0.531 |
| Specialized Survivor Clinic Cumulative |  |  | 0.910 | 0.871 | 0.951 | **<.0001** |
| Specialized Survivor Clinic Pre-1Yr | Yes vs No |  | 0.388 | 0.302 | 0.499 | **<.0001** |
| Family Physician Cumulative |  |  | 0.983 | 0.933 | 1.036 | 0.517 |
| Family Physician Pre-1Yr | Yes vs No |  | 0.858 | 0.662 | 1.113 | 0.249 |
| Radiation | Yes vs No |  | 0.698 | 0.525 | 0.928 | **0.013** |
| General Cancer Clinic Cumulative |  |  | 0.999 | 0.981 | 1.016 | 0.873 |
| General Cancer Clinic Pre-1Yr | Yes vs No |  | 1.081 | 0.838 | 1.394 | 0.550 |
| Rurality | Urban vs Rural |  | 0.753 | 0.497 | 1.140 | 0.180 |
| SES | 2 vs 1 |  | 0.948 | 0.671 | 1.341 | 0.764 |
|  | 3 vs 1 |  | 1.010 | 0.666 | 1.533 | 0.962 |
|  | 4 vs 1 |  | 0.999 | 0.688 | 1.451 | 0.997 |
|  | 5 vs 1 |  | 1.157 | 0.831 | 1.610 | 0.388 |
| Sex | Female vs Male |  | 1.024 | 0.806 | 1.300 | 0.849 |
| Surgery | Yes vs No |  | 0.661 | 0.520 | 0.839 | **0.001** |
| Treatment Before Index | Chemo and Chest Rad vs. Chemo only |  | 1.072 | 0.863 | 1.331 | 0.530 |
| Treatment Before Index | Chest Rad only vs. Chemo only |  | 0.428 | 0.341 | 0.538 | **<.0001** |

***Supplemental Table 7: Univariable Analysis for Transition State Non-Adherent to Adherent for Cardiomyopathy Risk (1 & 2 Year Surveillance Combined)***

| **Parameter** | **Class** | **RR** | **95% LCL** | **95% UCL** | **P<0.1** |  |
| --- | --- | --- | --- | --- | --- | --- |
| Age | Age at Follow-up Start | 0.844 | 0.777 | 0.917 | **<.0001** |  |
| BMT | Yes vs No | 1.378 | 0.906 | 2.095 | 0.134 |  |
| Cardiac Risk Year | 2 years vs 1 year | 1.860 | 1.394 | 2.482 | **<.0001** |  |
| Chemotherapy | Yes vs No | 1.633 | 1.006 | 2.653 | **0.047** |  |
| Chemotherapy + Radiation | Yes vs No | 2.087 | 1.541 | 2.826 | **<.0001** |  |
| Distance Nearest Specialized Survivor Clinic |  | 1.000 | 1.000 | 1.001 | 0.356 |  |
| Distance Nearest General Cancer Clinic |  | 1.000 | 0.998 | 1.001 | 0.772 |  |
| Specialized Survivor Clinic Cumulative |  | 1.198 | 1.127 | 1.274 | **<.0001** |  |
| Specialized Survivor Clinic Pre-1Yr | Yes vs No | 4.007 | 2.787 | 5.761 | **<.0001** |  |
| Family Physician Cumulative |  | 1.108 | 1.051 | 1.167 | **0.000** |  |
| Family Physician Pre-1Yr | Yes vs No | 1.304 | 0.983 | 1.730 | **0.065** |  |
| Radiation | Yes vs No | 1.887 | 1.352 | 2.635 | **0.000** |  |
| General Cancer Clinic Cumulative |  | 1.020 | 1.000 | 1.041 | **0.045** |  |
| General Cancer Clinic Pre-1Yr | Yes vs No | 1.139 | 0.834 | 1.554 | 0.413 |  |
| Rurality | Urban vs Rural | 1.239 | 0.790 | 1.944 | 0.350 |  |
| SES | 2 vs 1 | 1.112 | 0.747 | 1.656 | 0.601 |  |
|  | 3 vs 1 | 0.680 | 0.443 | 1.043 | **0.077** |  |
|  | 4 vs 1 | 0.866 | 0.574 | 1.305 | 0.490 |  |
|  | 5 vs 1 | 0.958 | 0.636 | 1.443 | 0.839 |  |
| Sex | Female vs Male | 1.733 | 1.300 | 2.310 | **0.000** |  |
| Surgery | Yes vs No | 1.746 | 1.243 | 2.453 | **0.001** |  |
| Treatment Before Index | Chemo and Chest Rad vs. Chemo only | 1.790 | 1.219 | 2.628 | **0.003** |  |
| Treatment Before Index | Chest Rad only vs. Chemo only | 3.083 | 2.128 | 4.466 | **<.0001** |  |

**APPENDIX 1: OHIP FEE BILLING CODES**

**OHIP billing codes used to capture family physician full history visits:**

A003, A004, A005, A006, A911, K131, A912, A265, A260, A662, A263, A264, A905, and A565

**OHIP billing codes used to identify breast imaging:**

Mammogram: OHIP Codes-X84-X185, J663, J863

Breast MRI: OHIP Code- X441

Breast ultrasound: OHIP Codes- J127, J427.

**OHIP billing codes used to identify echocardiogram occurrences:**

G560-G575
